# Nonlinear Phenomena in Biology and Medicine

**DOI:** 10.1155/2012/183879

**Published:** 2012-05-17

**Authors:** Vikas Rai, Sreenivasan R. Nadar, Ranjit K. Upadhyay

**Affiliations:** ^1^Department of Mathematics, Faculty of Science, Jazan University, 45142 Jizan, Saudi Arabia; ^2^National Institute of Mental Health, Bethesda, 20892 MD, USA; ^3^Indian School of Mines, 826004 Dhanbad, India

Nonlinear dynamics has changed our view of many biological phenomena; e.g., coexistence of consumer species competing for the same resource [1], biological pattern formation; e.g., clustering and differentiation of mesenchymal cells during pre-cartilage messenchymal condensation in chick limb chondrogenesis (bone patterning and formation) [2] and analysis of heart rate variability in healthy as well as diseased subjects [3] to name a few. The diversity of disciplines and successes of approaches based on non-linear dynamics, complexity theory, and systems biology in resolving a few difficult issues and answering some outstanding questions in recent years led to the idea of compiling a special volume on the subject.

 This special issue is dedicated to nonlinear phenomena in biology and medicine. The research papers which appear in this issue can be classified into two categories: (1) papers which discuss approaches to nonlinear time series analysis based on dynamical systems theory and (2) others which present models and their potential application to human behavior, biology and medicine. 

 In this issue, K. Friston and F. Ao draw attention of researchers in neuroscience to the possibility that action and perception can be understood in terms of minimization of a free energy surface functional which minimizes “sensory surprise”. Free energy is an information theoretic measure that bounds the surprise on sampling data, given a generative model. Authors compare two different approaches to describe agent action and prediction based on the free energy principle and optimal control reinforcement learning. In the first case, an agent's behavior is controlled by a free energy surface functional that minimizes “sensory surprise” along the trajectory in the phase space of a nonautonomous system with strong fluctuations. The mathematical definition of surprise is conditional entropy. In the last case, an agent's adaptive behavior is determined in order to maximize a reward.

 Epilepsy is the principal brain dysfunction which affects about 1% world population and has important public health implication. The conventional signal analysis methods such as the count of focal spike density, the frequency coherence, or spectral analyses are not reliable predictors. In this issue the paper by T. Khoa et al. describes a method based on fractal dimension as a principal tool to diagnose epilepsy. Their method combines independent component algorithm with averaging filter at the preprocessing step. The authors show that this improved method could be used to analyze EEG signals to diagnose epilepsy.

B. Francesco et al. review the applications of linear and nonlinear indexes in heart rate variability and show how these indexes are useful in clinical practice using data from patients.

 D. Vasco presents a contribution which focuses on a Bayesian Markov Chain Monte Carlo method to infer parameters in simple SIR model in epidemiology. Application of Markov chain models in conjunction with SSA is not new. The interesting part of the paper is that the author has successfully employed this method to measles and pertussis epidemic time series data from 60 UK cities. 

 K. Mohan presents a simulation model to understand the spread and control of lesions based on a planar graph representation for the central nervous system. The author demonstrates that the model is capable of generating a wide variety of lesion growth and arrest scenarios.

 A. Bidhendi and R. Korhonen contribute a paper on nonlinear deformations of cells during micropipette aspiration procedure to measure its viscoelastic properties. The paper examines a neo-Hookean model for micropipette aspiration. A fillet radius is considered at the opening of the micropipette to study its effect on the modeled response of the cell. The authors estimate optimal parameters of the model from the experimental stem cell data. Their findings suggest that the compressibility and bulk relaxation/fluid flow play a significant role in the deformation behavior of single cells and should be taken into account in the analysis of the mechanics of cells.

C. Pradhan et al. examine the fundamental nature of the brain electrical activities recorded as electroencephalogram (EEG). Linear stochastic models and spectral estimates are the most common methods for the analysis of EEG because of their robustness, simplicity of interpretation, and apparent association with rhythmic behavioral patterns in nature. The paper extends the application of higher-order spectrum in order to clarify the hidden characteristics of EEG signals that simply do not arise of random processes. This paper demonstrates the suitability of bispectral analysis to distinguish chaotic systems from filtered noises and normal background EEG activity. 

In sum, we note that these contributions present state of the art of their respective subdisciplines. The research papers appearing in this special issue will serve as a guide to what is yet to follow in this fascinating field of biology and medicine. Procedures laid down in the research papers by T. Khoa et al. and by C. Pradhan et al. can be combined to design a protocol for the diagnostics of epileptic disorders. We hope that this volume will serve interests of researchers working in the field of applied biology and medicine. 

*Vikas Rai* *Sreenivasan R. Nadar* *Ranjit K. Upadhyay*
